# Aquatic Therapy in Children and Adolescents with Disabilities: A Scoping Review

**DOI:** 10.3390/children11111404

**Published:** 2024-11-20

**Authors:** Anna Ogonowska-Slodownik, Oliwia Jakobowicz, Lyndsay Alexander, Andresa R. Marinho-Buzelli, Catherine Devion, Natalia Morgulec-Adamowicz

**Affiliations:** 1 Faculty of Rehabilitation, Jozef Pilsudski University of Physical Education in Warsaw, 00-968 Warszawa, Poland; oliwia.jakobowicz@awf.edu.pl (O.J.); natalia.morgulec@awf.edu.pl (N.M.-A.); 2 KITE Research Institute, Toronto Rehabilitation Institute—University Health Network, Toronto, ON M5G 2A2, Canada; 3 Scottish Centre for Evidence-Based, Multi-Professional Practice: A JBI Centre of Excellence, School of Health Sciences, Robert Gordon University, Aberdeen AB10 7AQ, UK; l.a.alexander@rgu.ac.uk; 4 St. John’s Rehab Research Program, Sunnybrook Research Institute, Toronto, ON M4N 3M5, Canada; andresa.marinho@mail.utoronto.ca; 5 Library Services, Sunnybrook Health Sciences Centre, Toronto, ON M4N 3M5, Canada; catherine.devion@sunnybrook.ca; 6 Disability Resource Center, University of Arizona, Tucson, AZ 85721, USA

**Keywords:** child, teenagers, pediatric, aquatic physical therapy, water-based

## Abstract

Globally, around 1 in 10 children aged 0–17 years have moderate-to-severe disabilities. The aquatic environment provides hydrostatic and hydrodynamic characteristics that make exercise and therapy feasible for children and adolescents with disabilities. The objective of this scoping review is to understand the extent and type of evidence in relation to the use of aquatic therapy in children and adolescents with disabilities. The eligibility criteria were as follows: participants—children and/or adolescents with disabilities aged from 6 to 18 years old; concept—aquatic therapy interventions; context—any available setting. The databases searched included MEDLINE, CINAHL, EMBASE, PsycINFO, AMED, Eric, Scopus, Web of Science, Epistemonikos, and one register, Cochrane Central Register of Controlled Trials. In total, 52 reports met the inclusion criteria. Most of the studies included children/adolescents with autism spectrum disorder (ASD; 46.7%)—442 participants in 21 studies in total. The majority of interventions were based on aquatic exercise (35%). Most often, interventions were conducted for 8 weeks, with 2 sessions a week lasting 60 min. The most common type of intervention for children and adolescents with ASD and Down syndrome was swimming. Participants with attention deficit hyperactivity disorder, neuromuscular disorders, and cerebral palsy were more often treated with aquatic exercises. This scoping review could guide practitioners, clinicians, and researchers on what type, setting, and content of aquatic therapy interventions, including exercise types, intervention duration, number of sessions, frequency, facility, and provider, are used with children and adolescents with disabilities.

## 1. Introduction

Globally, the United Nations Children’s Fund (UNICEF) estimates that nearly 240 million children aged 0–17 years have moderate-to-severe disabilities, which accounts for 1 in 10 of all children worldwide [1]. According to the World Health Organization (WHO), disability has three dimensions [2]: impairment in a person’s body structure or function, or mental functioning (loss of a limb, loss of vision or memory loss); activity limitation (difficulty seeing, hearing, walking, or problem solving) and participation restrictions in normal daily activities (learning, playing, engaging in social and recreational activities). According to the Convention on the Rights of Persons with Disabilities (CRPD), children with disabilities “include those who have long-term physical, mental, intellectual or sensory impairments which in interaction with various barriers may hinder their full and effective participation in society on an equal basis” [3].

Ensuring access to high-quality services in pediatric physiotherapy is important to respond to the diverse needs of children [4]. The aim of pediatric physiotherapy is to minimize the impact of impairments on the child and to enhance the overall quality of life for the child and the family [5]. The Academy of Pediatric Physical Therapy advocates that pediatric therapy should assist each child in achieving their maximum potential for independent function and to advance partnership in home, school, and society surroundings [6].

Aquatic exercise is recommended for healthy individuals as well as people with various health problems [7]. Water, as opposed to land, is sometimes the only place where movement can be performed by people with severe disability [8]. The hydrostatic and hydrodynamic characteristics of the water make exercise feasible for children with disabilities [9]. Movement in the water, for those who are in pain or have problems with physical functioning, is possible thanks to buoyancy which gives support and unloads the joints.

Aquatic therapy is defined by Medical Subject Headings as “physical therapy administered while the body is immersed in an aquatic environment”. Based on research, aquatic therapy has been proven to have a positive impact on the blood fat levels, health-related physical fitness, and immune functions of children with disabilities [10]. Aquatic therapy reduces muscle tension, increases the ability of soft tissues to stretch [11], and has a positive effect on gross motor skills in children with cerebral palsy (CP) [12]. Research indicates that aquatic therapy sessions lead to children feeling happy, relaxed, and calm, and enable them to participate in other activities in school [13]. The hydrostatic pressure provided by water improves the efficiency of the cardiorespiratory system in children with respiratory problems [14]. In children with autism spectrum disorder (ASD), significant improvements in physical competencies and functioning in school have been observed [15]. Aquatic therapy has been found to have a positive effect on respiratory function, postural control, and overall functioning in children with Duchenne dystrophy [16]. For children with disabilities, the social part of group aquatic therapy with their peers without disabilities is also very important. Research showed that an aquatic after-school program led to improved acceptance and overall quality of life [17].

A preliminary search of PROSPERO, MEDLINE, the Cochrane Database of Systematic Reviews, and JBI Evidence Synthesis was conducted. No current or ongoing systematic or scoping reviews have been identified to date that have mapped the evidence related to aquatic therapy for children and adolescents with disability. Previous systematic reviews have focused on analyzing the effects of aquatic intervention on gross motor skills [12] and aquatic intervention based on the Halliwick concept of psychomotor development, gross motor function, and aquatic skills in children with CP [18]. In children with ASD, the literature was systematically reviewed in terms of the use of aquatic therapy as a treatment of social and behavioral aspects [19]. The effectiveness of aquatic therapy on motor and social skills as well as executive function was also assessed in children with neurodevelopmental disorders [20]. One review quantified and summarized the various aquatic interventions in children with disabilities, but was conducted over 10 years ago [21]. None of the recent reviews, to our knowledge, have comprehensively or specifically looked at the use of aquatic therapy in children and adolescents with all types of disability.

The objective of this scoping review is to assess the extent of the literature in the field of aquatic therapy for children and adolescents with disabilities.

Review questions

1. What conditions and/or disabilities may be treated and managed with aquatic therapy in children and adolescents?
2. What is the content of aquatic therapy interventions reported including exercise types, intervention duration, number of sessions, and frequency?
3. What benefits and risks are reported for aquatic therapy in children and adolescents with disabilities?
4. What are the types and settings of aquatic therapy interventions for specific conditions and/or disabilities in children and adolescents?

## 2. Materials and Methods

The proposed scoping review was conducted in accordance with the JBI methodology for scoping reviews [22]. The review protocol has been registered in Open Science Framework (DOI 10.17605/OSF.IO/927UP).

### 2.1. Eligibility Criteria

The Participant, Concept, Context (PCC) approach for developing eligibility criteria was adopted.

#### 2.1.1. Participants

This review included evidence sources where participants were children or adolescents aged from 6 to 18 years old and living with disabilities. Age and disability were considered as eligibility criteria. We included all articles where the mean age of the participants was between 6 and 18 years old. Articles describing children and adolescents with and without disabilities were only included when data were reported separately for these groups. To explore the variety of individuals with disability who may participate in aquatic intervention, the United Nations Convention on the Rights of Persons with Disabilities [3] definition of people with disability was adopted. Children and adolescents with congenital and acquired disability were included.

Evidence sources with participants who are young children (from 0 to 5 years old), or children or adolescents without disabilities were excluded.

#### 2.1.2. Concept

This review aimed to identify the available literature on aquatic therapy interventions. Examples of aquatic therapy interventions included Halliwick [18], therapeutic exercises (e.g., strength, flexibility), breathing exercises, activities of daily living training, swimming and relaxation. The intervention could be group-based or individual. This review also aimed to understand aquatic therapy intervention delivery methods for children and adolescents (e.g., types and settings of interventions, benefits and risks, and suitability for specific conditions and/or disabilities). Studies were excluded if they only mentioned aquatic therapy but did not describe specific interventions. Passive use of aquatic settings such as immersion, balneotherapy, and swimming with dolphins were excluded.

#### 2.1.3. Context

The context included any setting such as primary care, secondary care, or community locations. No limitations on the country where the intervention was conducted were adopted.

#### 2.1.4. Types of Sources

This scoping review considered all study designs including experimental, exploratory, and descriptive study designs. In this review, we focused on primary research articles. Non-original works (i.e., reviews, guideline documents, editorials, viewpoints, letters to the editor, abstracts) were excluded.

### 2.2. Search Strategy

The search strategy aimed to locate all published studies describing aquatic therapy for children and adolescents with disabilities. The text words contained in the titles and abstracts of relevant articles and the index terms used to describe the articles were used to develop a full search strategy, in consultation with an academic librarian (see Supplementary Materials for an example of the full search strategy for MEDLINE). The search strategy, including all identified keywords and index terms, was adapted for each included database, and the search was performed by the academic librarian on the 16 February 2024. The databases searched included MEDLINE (Ovid), CINAHL (EBSCOHost), EMBASE (Ovid), PsycINFO (Ovid), AMED (Ovid), Eric, Scopus, Web of Science, Epistemonikos, and one register, the Cochrane Central Register of Controlled Trials (Ovid). The reference lists of all included sources of evidence were screened for additional studies to be considered for inclusion. Additional articles were manually found via the personal collections of the authors. We focused on published literature. 

Studies published in any language that the review team could access a translation through deepl.com translation software (https://www.deepl.com/pl/translator, accessed on 15 August 2024) were included. Studies published since 2012 were included as there was a systematic review on a similar topic published in 2013 [21], in which the search was limited to 2012. This enabled the authors to focus on the most recent evidence since the publication of the previous review.

### 2.3. Study/Source of Evidence Selection

Following the search, all identified citations were collated and uploaded into EndNote 20 (Clarivate Analytics, Philadelphia, PA, USA) and duplicates removed. Following de-duplication, citations were uploaded to Covidence (Veritas Health Innovation, Melbourne, Australia) to facilitate screening and data extraction. Titles and abstracts were screened independently by two reviewers for assessment against the inclusion criteria. Any disagreement at this point was solved by consulting a third reviewer. Potentially relevant records were retrieved in full and their citation details imported into Covidence. The full text of selected articles was assessed in detail against the inclusion and exclusion criteria by two independent reviewers. Reasons for the exclusion of sources at full-text screening were recorded and reported in the scoping review. Any disagreements that arose between the reviewers at the full-text screening stage were resolved through discussion and consultation with a third reviewer. The results of the search and the reasons for excluding studies are reported in full and presented in a Preferred Reporting Items for Systematic Reviews and Meta-analyses extension for scoping review (PRISMA-ScR) flow diagram [23].

### 2.4. Data Extraction

Data were extracted from records included in the scoping review by two independent reviewers using Covidence (Veritas Health Innovation, Melbourne, Australia). Specific details about the participants, concept, context, and benefits/risks relevant to the review questions were extracted. The data extraction tool was piloted by two reviewers on two randomly selected studies. Any disagreements that arose between reviewers were resolved through discussion and consulting an additional reviewer.

### 2.5. Data Analysis

A descriptive analysis of data was conducted following the conceptual categories related to the review questions. The Template for Intervention Description and Replication (TIDieR) checklist was used to map the aquatic therapy interventions regarding how, where, and to whom aquatic therapy was provided [24]. A qualitative content analysis approach was adopted to analyze benefits and risks [25]. An inductive approach was used to identify categories related to benefits as well as risks and precautions when treating children and adolescents in aquatic settings. The benefits were grouped into the following domains: functional performance, physiological domain, psychosocial domain, and aquatic skills [26]. Review data are presented in tables, figures, and narrative summaries of findings relevant to the review aim and questions.

## 3. Results

**Figure 1:**
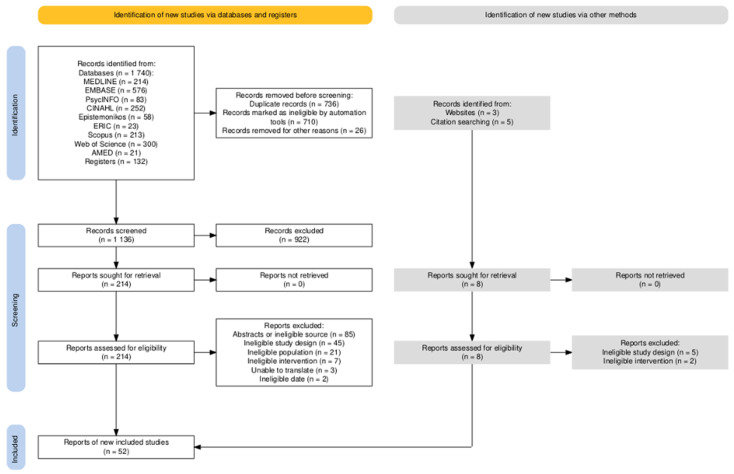
PRISMA flow chart [27].

The database search revealed 1872 records, and another 8 were found through other sources (Figure 1). After excluding duplicates using Covidence (Veritas Health Innovation, Melbourne, Australia) and manually, 1136 records were screened based on the title and abstract. Full-text versions of 222 reports were assessed for eligibility. The main reasons for the exclusion of a report at full-text review included wrong study design (n = 130), wrong population (n = 21), and wrong intervention (n = 7). Finally, 52 reports met the inclusion criteria. Each report represented separate studies; therefore, we used the term report and study interchangeably.

**Figure 2:**
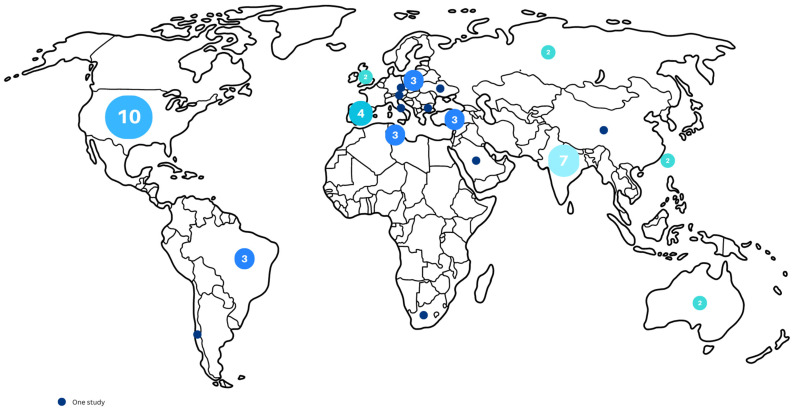
Map with the number of studies published in different countries.

Most of the studies (69%) were published after 2020 (n = 36) and 33% (n = 17) were conducted in Europe (Figure 2). In total, 25% of studies (n = 12) were conducted in Asia and all studies from North America (19%) originated in the US (n = 10). Two articles did not provide information on where the research was conducted [28,29].

In the majority of the included studies (88%), an experimental research design was applied. In twenty-one studies, a randomized control trial design was used [28,30,31,32,33,34,35,36,37,38,39,40,41,42,43,44,45,46,47,48,49], thirteen studies used a single-subject design [29,50,51,52,53,54,55,56,57,58,59,60,61], ten studies adopted a non-randomized experimental design [11,15,62,63,64,65,66,67,68,69], and two reports were feasibility studies [70,71]. The remaining studies included descriptive research, four case studies [13,72,73,74], and two qualitative studies [75,76].

### 3.1. Characteristics of Conditions and/or Disabilities Treated and Managed with Aquatic Therapy

**Figure 3:**
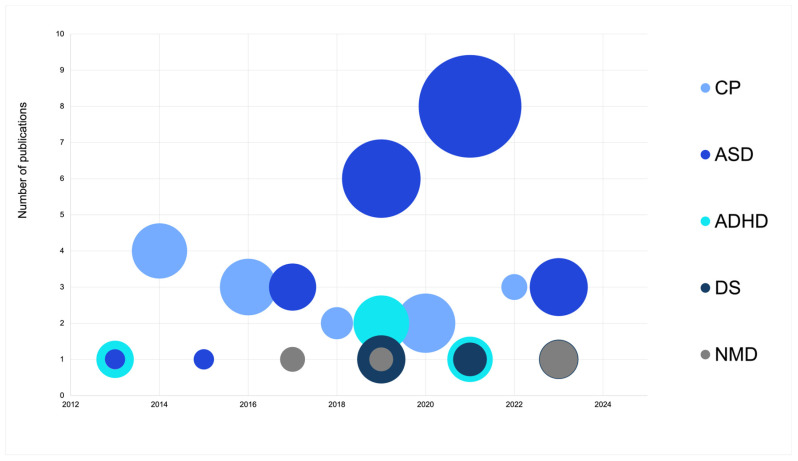
Number of publications, number of participants, and types of disabilities published since 2012. Note: The size of the bubbles represents the number of participants; ASD—autism spectrum disorder; ADHD—attention deficit hyperactivity disorder; CP—cerebral palsy; DS—down syndrome; NMD—neuromuscular disorder.

Most of the studies included children/adolescents with ASD (46.7%), representing 442 participants and 21 studies in total (Figure 3). There were 15 studies with 230 participants with CP. The rest of the studies included participants with attention deficit hyperactivity disorder (ADHD) (four studies, 127 participants), Down syndrome (DS) (three studies, 97 participants) and neuromuscular disorders (NMD) (three studies, 51 participants). Other studies included children/adolescents with juvenile idiopathic arthritis, mental health disorders, obesity, neurodevelopmental disorders, scoliosis, and intellectual disability.

### 3.2. Content of Aquatic Therapy

**Figure 4:**
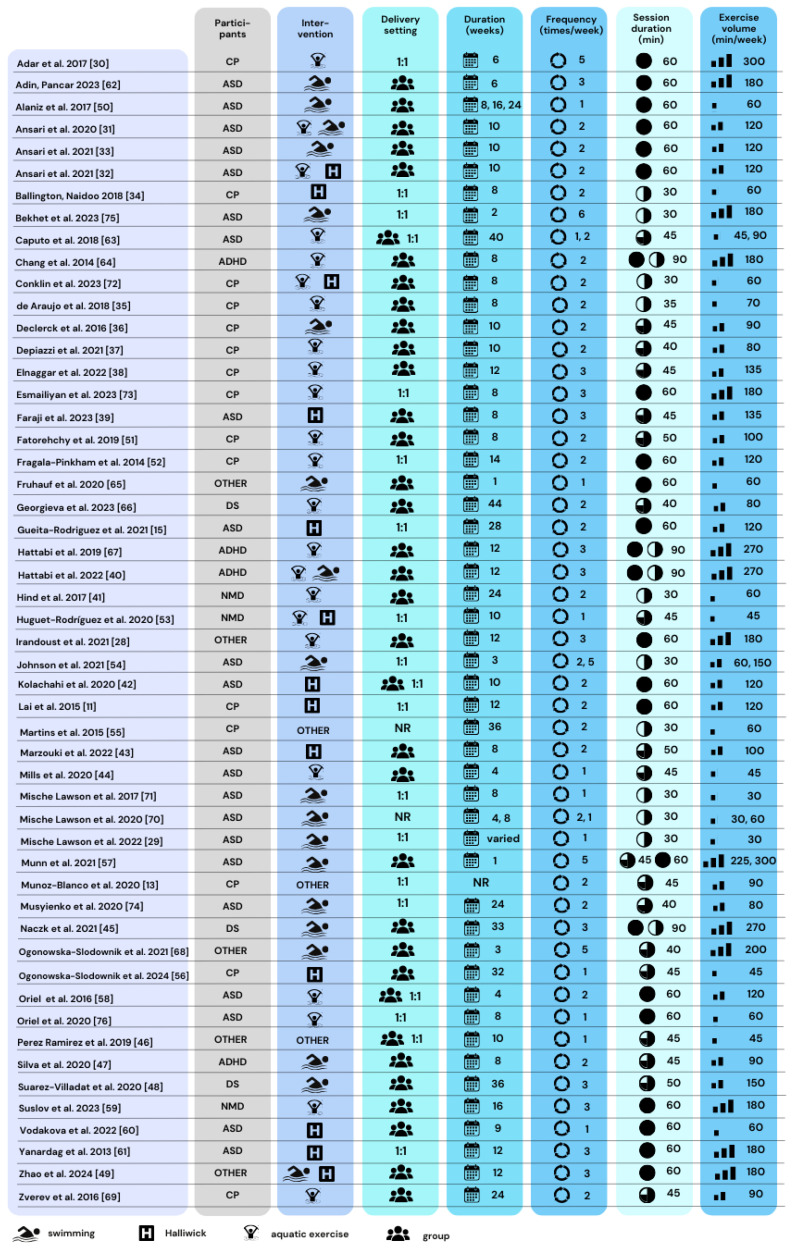
Characteristics of the aquatic interventions. ASD—autism spectrum disorder; ADHD—attention deficit hyperactivity disorder; CP—cerebral palsy; DS—down syndrome; NMD—neuromuscular disorder; NR—not reported. [11,13,15,28,29,30,31,32,33,34,35,36,37,38,39,40,41,42,43,44,45,46,47,48,49,50,51,52,53,54,55,56,57,58,59,60,61,62,63,64,65,66,67,68,69,70,71,72,73,74,75,76,77].

The included papers’ analyses revealed varied water intervention types and very diverse styles of intervention protocol descriptions (Figure 4). In 40% (n = 21) of the analyzed interventions, the content was presented without a timeline of the session (therapeutic unit). Overall, the content of the aquatic therapy interventions reported in the included studies was grouped into five types, which were not mutually exclusive and combined different types of physical activities (e.g., water walking and water breathing exercises). The first type (35%, n = 18) was interventions based on aquatic exercise including water walking and exercises (n = 5) [28,51,59,66,69], aerobic exercise in water (n = 5) [30,37,44,52,64], exercise in water with aquatic games (n = 5) [58,63,67,76,77], trunk exercises (n = 2) [35,73], and plyometric exercises (n = 1) [38]. The second type of intervention (31%) was based mainly on swimming [29,32,36,45,47,48,50,54,57,62,65,68,70,71,74,75]. The third type of intervention (17%) referred to the Halliwick concept [11,15,34,39,42,43,56,60,61]. The fifth type was ‘mixed’ (12%), combining aquatic exercises with swimming [31,40] or the Halliwick concept [33,53,72], and swimming with the Halliwick concept [49]. Three other studies included specific techniques and methods such as Watsu, Bad Ragaz, and Halliwick [46,55] and craniosacral therapy [13].

The most frequent type of water intervention was group classes (59.6%), and one-on-one therapy was chosen by 28.9% of researchers. Two studies [55,70] did not specify the delivery setting. Interventions in water lasted from 1 to 40 weeks, although the most common duration was 8 weeks (25%) [34,35,39,43,47,50,51,64,70,71,72,73,76]. Interventions were conducted from one to five times a week, with most often being twice a week (50%) [11,13,15,31,32,33,34,35,36,37,42,43,47,51,52,55,58,63,64,66,69,70,72,74,77]. The duration of a single intervention ranged from 30 to 90 min, with the most frequent being 60 min (38%) [11,15,28,30,31,32,33,42,49,50,52,58,59,60,61,62,65,73,76]. The exercise volume of the intervention ranged from 30 to 300 min/week, with the most frequent being 60 min/week.

The training intensity of the intervention was mentioned in 13 studies (25%). Five studies defined the intensity based on percentage of heart rate [28,37,40,52,59]. Four studies mentioned the intensity to be “moderate” [49,64,67,72] and one “moderate-to-vigorous” [63]. Another three studies described the intensity as “high” (best effort) [38], one study stated that it was individually set [69], and one that it varied between the participants [48].

The water temperature was described in 22 out of 52 studies. A temperature below 30 °C was set in ten studies [31,32,36,40,44,49,60,66,68,69]. Nine studies reported a temperature between 30 °C and 33 °C [30,35,37,38,52,56,59,72,76]. Three studies reported using water with a temperature in the range of 33–36 °C [11,51,77]. The specific submersion level was provided in four studies [38,51,52,72]. Nine studies provided the depth of the pool in which the intervention was carried out [44,45,49,56,59,66,68,69,76].

### 3.3. Benefits and Risks of Aquatic Therapy

Most studies (n = 22) reported benefits in the functional performance domain in the subdomain motor performance (n = 16) (Table 1). Benefits in the psychosocial domain were reported in 18 studies, with the most common improvement being seen in behavior (n = 7). Physiological benefits were reported in 11 articles, showing improvements mostly in pulmonary function, sleep, and body composition. Benefits in aquatic skills were reported in six studies. Two studies did not report benefits for the participants as they were focused on the feasibility of the interventions [37,77]. One study reported benefits that were not consistent between the participants [29].

Only nine studies reported risks associated with aquatic interventions. Most of them were minor adverse events like muscular pain or muscle tension [59], blisters [52], fatigue [77], stubbed toes, stomach pain [37], or bruising [44]. Four studies reported no adverse effects during the study [13,15,48,53].

### 3.4. Types of Aquatic Therapy Interventions for Specific Conditions and/or Disabilities

**Figure 5:**
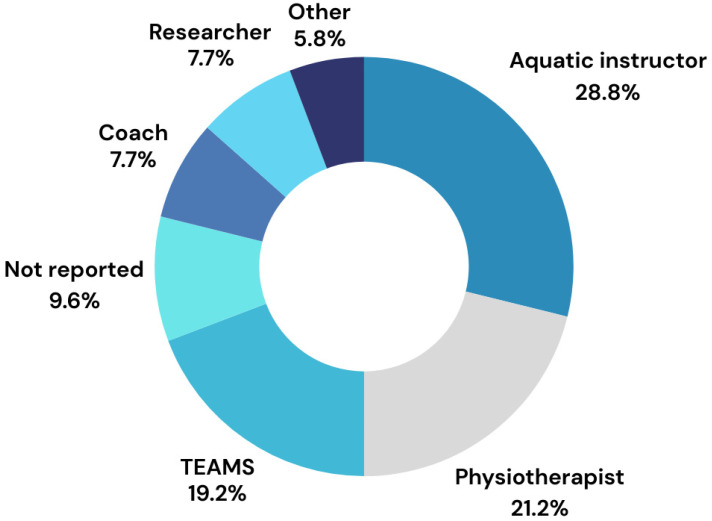
Specialists delivering aquatic interventions.

The analyzed aquatic therapy interventions reported in the studies were delivered by a wide variety of specialists (Figure 5). Most interventions (29%) were delivered by water instructors including certified swimming trainers/coaches who had previous experience in training children with special needs [31,32,33,42,43,49,57], or swimming instructors [54,60,64,66,70,75], swimming trainers/coaches without additional experience [48], and a specialist in hydro rehabilitation [59]. In 11 studies (21%), the interventions were provided by physiotherapists with experience in pediatrics [13,35,38,46,72,77] and/or aquatic therapy [11,15,30,52,53]. Ten interventions were delivered by teams composed of at least two people from the following groups: researchers, physiotherapists, exercise specialists, certified adapted physical activity experts, recreational therapists, occupational therapists, certified multi-systemic aquatic therapy (CI-MAT) experts, swimming instructors, swimming trainers, experienced lifeguards, exercise physiologists, students, trained volunteers, and/or parents/guardians. Five studies did not report who delivered the intervention [28,47,58,62,74].

Eighteen studies (35%) reported that the intervention was conducted at a community pool (including university swimming pools) [31,33,36,42,43,44,45,47,54,61,62,63,64,65,69,73,75,76]. The outpatient clinic pool served as the intervention context for seven studies [15,38,49,50,51,55,77], and a hospital for one study [37]. Furthermore, five studies did not provide enough details on the specific setting [13,35,39,40,66]. Finally, 21 studies (40%) did not report the context in which the water intervention occurred.

**Figure 6:**
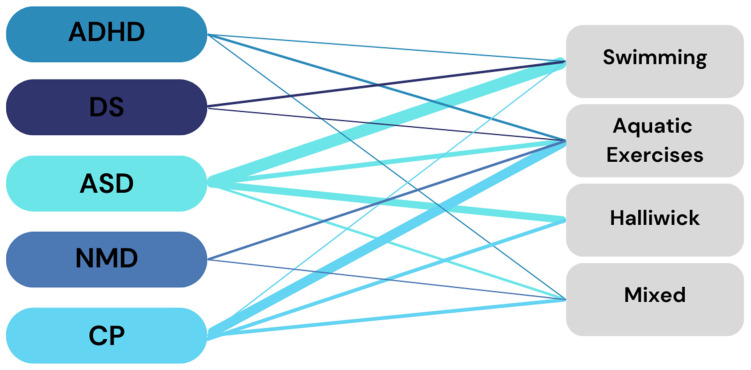
Type of disability and the type of intervention. Note: Thicker line represent more studies; ASD—autism spectrum disorder; ADHD—attention deficit hyperactivity disorder; CP—cerebral palsy; DS—down syndrome; NMD—neuromuscular disorder.

The most common type of intervention for children and adolescents with ASD and DS was swimming (Figure 6). Participants with ADHD, NMD, and CP were more often treated with aquatic exercises.

## 4. Discussion

This scoping review sought to identify the extent of the literature in the field of aquatic therapy for children and adolescents with disabilities. A recent acceleration in the research in this area was observed as 69% of the studies were published after 2020. In addition, 21 (40%) of the analyzed studies were RCTs, which is different from the results obtained by Karklina et al. [21], who found only 11 RCTs out of 45 analyzed studies in the previous systematic review.

Analyzing the type of disability treated with aquatic therapy, we saw studies with participants with ASD emerging in research in recent years (22 studies in total) compared to the previous review [21] with 9 studies. This might also be due to the increasing trend in the prevalence of ASD in children and adolescents [78]. The increase in diagnosis of ASD in recent years is often explained by a broader definition of ASD, changes in diagnostic criteria and screening tools, shifts in research methods, and increased awareness of ASD [78]. This increase in children and adolescents with ASD treated with aquatic therapy is also confirmed by the systematic reviews which were published recently analyzing other therapeutic interventions in this group [79,80]. The second most common type of disability was CP, which is similar to previous results. The third group most visible in this review is children and adolescents with ADHD, who were present in four studies in the last decade, but not reported in the previous review by Karklina et al. [21]. This could be an effect of the high international prevalence of ADHD in children and adolescents (8%), relatively comparable across the globe, revealed in an umbrella review of meta-analyses by Ayano et al. [81] and a consistent rise in recent years [82].

We observed a large variability in terms of the sample sizes, which did not depend on the type of disability. The studies including children with CP ranged from 1 [72] to 56 participants [38]. The sample size for children/adolescents with ASD ranged from 3 [29,61,74] to 86 [57], while for ADHD, it ranged from to 27 [63] to 40 [39,66]. This scoping review revealed that recent scientific evidence on aquatic therapy for children/adolescents with disabilities primarily focused on individuals with ASD and CP, which confirms the trend noticed by Karklina et al. [21]. Although other conditions and disabilities were present, those were only single studies, and the spectrum of condition types was limited compared to Karklina et al.’s review from 2013 [21].

We faced the same problem as that stated in the previous review [21] with defining the content of the aquatic interventions. The researchers also pointed out the problems with parameters that are not clear [12]. No common guidelines and definitions for the different interventions are available, and various intervention types were used within one program. Most of the studies analyzed in this scoping review did not report the training intensity of the intervention. One reason might be the difficulty of measuring the intensity in the water, but the other might be that the interventions were focused on performing specific exercises rather than the intensity. Interestingly, none of the interventions which were based on swimming described the intensity. Water temperature was reported in less than half of the studies despite it being crucial for rehabilitation use in children [83]. Interestingly, only three studies used water in the range of neutral temperatures for the body (33.5–35.5 °C), which is most commonly used for aquatic therapy [9]. Aquatic professionals should consider the type of disability, intensity, and duration of the exercise based on the water temperature [21].

In the context of all the publications involved in this review, the most common intervention duration was 8 weeks with twice-weekly periodization. There was one intervention lasting as long as 44 weeks involving water exercises, which showed significant improvement in praxis function in children with DS [66]. However, Munn et al. [57] reported benefits after just a 5-day adapted swimming program. Considering the variability in the interventions, there is a need to include follow-up measurements to test if the benefits are maintained over time, after the end of the intervention.

The aquatic environment provides benefits that would not be available on land, including positive effects on motor function, muscle strength, and increased physical performance. It is also a playful environment filled with possibilities for improving the sensory system [34]. The type of intervention was chosen mainly based on the specific needs of the study participants and the desire to provide them with adequate benefits. Other systematic reviews reported similar benefits like improvements in mental health, balance control, and independent movement in water [20]. The Halliwick concept used for children with CP was shown to be effective in improving gross motor function, aquatic skills, and social interaction skills [18]. Although only 9 out of 52 studies monitored risks/adverse events, it seems that the potential benefits of aquatic therapy interventions for children and adolescents may outweigh possible risks and minor adverse events. This could be an effect of the trained and experienced intervention providers present in the majority of the analyzed studies. The other reason might be that the aquatic setting provides a safe environment thanks to the properties of water.

The TIDieR checklist was used to map the aquatic therapy interventions regarding how, where, and to whom aquatic therapy was provided [24]. The accurate and detailed reporting of research is critical if the research being described is to be trusted and useful. Even though twenty-one studies incorporated a randomized controlled trial design, which is considered to be the highest level of evidence, most of the studies were missing some of the descriptors, which would allow the studies to be replicated and the recommendations to be reliably implemented in the clinical setting.

In the majority of studies, the aquatic interventions were delivered in a group setting, either by aquatic instructors or coaches. Participants exercising in groups were mainly individuals with ASD and ADHD. Physiotherapists mainly worked individually (one-on-one) during aquatic interventions with children and adolescents with CP. Interestingly, types of aquatic interventions (swimming, aquatic exercises, Halliwick concept, mixed) were not specific to a group of providers.

Regarding types of aquatic therapy interventions specific for particular conditions and/or disabilities in children and adolescents, it appeared that all types of aquatic interventions (swimming, aquatic exercises, Halliwick, and mixed) were provided for individuals with CP and ASD with dominant participation in aquatic exercises and swimming, respectively. For individuals with ADHD, the most common were aquatic exercises, followed by swimming and mixed interventions. Children and adolescents with DS were usually provided with swimming and aquatic exercises, while individuals with NMD participated in aquatic exercises and mixed interventions. Such variety in the types of aquatic interventions applicable for various conditions/disabilities seems to be promising for practitioners and clients, who may choose and change them in the case of long-lasting treatment.

### Limitations and Strengths

One of the limitations of this review relates to the scope of the search, which was limited to peer-reviewed articles. Only articles published in English and Spanish were included as there was no tool available which would translate, for example, Arabic or Korean articles reliably. Most of the analyzed studies did not describe exercise intensity and did not include follow-ups. While the scoping review achieved its objective of mapping the evidence, there are specific areas that require further investigation to enhance clinical applicability and understanding of the sustainable effects of aquatic therapy in children and adolescents with disabilities.

The strengths of this review include the rigorous and transparent approach taken to select relevant articles, and the reporting of the review, conducted in accordance with JBI methodology for scoping reviews [22] and the PRISMA-ScR guidelines [23]. The strengths also lie in the collaboration of a multidisciplinary team of reviewers, comprising clinicians and researchers who have experience in aquatic therapy, academics with expertise in methodology, and a librarian.

## 5. Conclusions

This scoping review provides an updated, evidence-based summary of the literature in the field of aquatic therapy for children and adolescents with disabilities. 

The observed conditions and/or disabilities treated and managed with aquatic therapy in children and adolescents analyzed in this review revealed a broad group of aquatic therapy beneficiaries among children and adolescents with disabilities. However, emerging interest in two groups of participants with ASD and ADHD was noticed in recent research, which seems to be in accordance with the increased international prevalence of ASD and ADHD in children and adolescents.

The outcomes of this review confirmed a wide range of benefits of aquatic therapy for children and adolescents with disabilities mainly in the functional performance and psychosocial domains, with a focus on motor performance enhancement.

This scoping review also identified various gaps in the evidence of aquatic therapy interventions. It is recommended that future studies on aquatic therapy programs include more detail on intervention content and explicitly address the risks. 

This scoping review could guide practitioners, clinicians, and researchers on what type, setting, and content of aquatic therapy interventions, including exercise types, intervention duration, number of sessions, frequency, facility, and provider are used with children and adolescents with disabilities. Such overviews may help them to develop aquatic intervention programs for particular groups of individuals with special needs.

## Figures and Tables

**Table 1:** Domains of reported benefits.

Domain	Benefits
Functional performance	Motor performance [11,30,34,35,39,43,45,47,49,51,52,55,59,60,61,72] Functional performance [38,53,66] Balance [33,38,69] Strength [73]
Physiological domain	Body composition [28,42,45,48,70] Sleep [32,58,71] Pulmonary function [28,53,62,68]
Psychosocial domain	Behavior [31,40,54,64,70,74,76] Social skills [15,43,63,65] Cognitive function [13,40,67] Mental health [44,47] Academic [40] Enjoyment [11,13,56] Health-related quality of life [30,46]
Aquatic Skills	Core aquatic skills [56,60,61,75] Swimming skills [36,57] Water safety [50]

## Data Availability

Available upon request directed to anna.ogonowskaslodownik@awf.edu.pl.

## Supplementary Materials

The following supporting information can be downloaded at: https://www.mdpi.com/article/10.3390/children11111404/s1, Table S1: Full Search Strategy for MEDLINE.

## References

[1] United Nations Children’s Fund (2021). Seen, Counted, Included: Using Data to Shed Light on the Well-Being of Children with Disabilities.

[2] World Health Organization (2001). International Classification of Functioning, Disability and Health (ICF).

[3] Convention on the Rights of Persons with Disabilities. https://social.desa.un.org/issues/disability/crpd/convention-on-the-rights-of-persons-with-disabilities-articles.

[4] Camden C., Mulligan H., Cinar E., Gauvin C., Berbari J., Nugraha B., Gutenbrunner C. (2023). Perceived strengths and weaknesses of paediatric physiotherapy services: Results from an international survey. Physiother. Res. Int..

[5] Hong C.S., Palmer K. (2003). Occupational therapy and physiotherapy for children with disabilities. J. Fam. Health Care.

[6] Academy of Pediatric Physical Therapy. https://pediatricapta.org/includes/fact-sheets/pdfs/FactSheet_ABCsofPediatricPT_2019.pdf?v=2.

[7] Faíl L.B., Marinho D.A., Marques E.A., Costa M.J., Santos C.C., Marques M.C., Izquierdo M., Neiva H.P. (2022). Benefits of aquatic exercise in adults with and without chronic disease-A systematic review with meta-analysis. Scand. J. Med. Sci. Sports.

[8] Conaster P., James E., Karabulut U. (2018). Adapted Aquatics for Children with Severe Motor Impairments. Int. J. Aquat. Res. Educ..

[9] Becker B.E. (2009). Aquatic therapy: Scientific foundations and clinical rehabilitation applications. PM&R.

[10] Kim K.H., Lee B.A., Oh D.J. (2018). Effects of aquatic exercise on health-related physical fitness, blood fat, and immune functions of children with disabilities. J. Exerc. Rehabil..

[11] Lai C.J., Liu W.Y., Yang T.F., Chen C.L., Wu C.Y., Chan R.C. (2015). Pediatric aquatic therapy on motor function and enjoyment in children diagnosed with cerebral palsy of various motor severities. J. Child Neurol..

[12] Roostaei M., Baharlouei H., Azadi H., Fragala-Pinkham M.A. (2017). Effects of Aquatic Intervention on Gross Motor Skills in Children with Cerebral Palsy: A Systematic Review. Phys. Occup. Ther. Pediatr..

[13] Muñoz-Blanco E., Merino-Andrés J., Aguilar-Soto B., García Y.C., Puente-Villalba M., Pérez-Corrales J., Güeita-Rodríguez J. (2020). Influence of Aquatic Therapy in Children and Youth with Cerebral Palsy: A Qualitative Case Study in a Special Education School. Int. J. Environ. Res. Public Health.

[14] Dimitrijević L., Aleksandrović M., Madić D., Okičić T., Radovanović D., Daly D. (2012). The effect of aquatic intervention on the gross motor function and aquatic skills in children with cerebral palsy. J. Hum. Kinet..

[15] Güeita-Rodríguez J., Ogonowska-Slodownik A., Morgulec-Adamowicz N., Martín-Prades M.L., Cuenca-Zaldívar J.N., Palacios-Ceña D. (2021). Effects of Aquatic Therapy for Children with Autism Spectrum Disorder on Social Competence and Quality of Life: A Mixed Methods Study. Int. J. Environ. Res. Public Health.

[16] de Lima A.A.R., Cordeiro L. (2020). Aquatic physical therapy in individuals with muscular dystrophy: Systematic scoping review. Fisioter. Pesqui..

[17] Oriel K.N., Marchese V.G., Shirk A., Wagner L., Young E., Miller L. (2012). The psychosocial benefits of an inclusive community-based aquatics program. Pediatr. Phys. Ther..

[18] Tapia C., Constanzo J., González V., Barría R.M. (2023). The Effectiveness of Aquatic Therapy Based on the Halliwick Concept in Children with Cerebral Palsy: A Systematic Review. Dev. Neurorehabilit..

[19] Mortimer R., Privopoulos M., Kumar S. (2014). The effectiveness of hydrotherapy in the treatment of social and behavioral aspects of children with autism spectrum disorders: A systematic review. J. Multidiscip. Healthc..

[20] Shariat A., Najafabadi M.G., Dos Santos I.K., Anastasio A.T., Milajerdi H.R., Hassanzadeh G., Nouri E. (2024). The Effectiveness of Aquatic Therapy on Motor and Social Skill as Well as Executive Function in Children With Neurodevelopmental Disorder: A Systematic Review and Meta-analysis. Arch. Phys. Med. Rehabil..

[21] Karklina B., Declerck M., Daly D.J. (2013). Quantification of Aquatic Interventions in Children With Disabilities: A Systematic Literature Review. Int. J. Aquatic Res. Educ..

[22] Peters M.D.J., Godfrey C., McInerney P., Munn Z., Tricco A.C., Khalil H., Aromataris E., Munn Z. (2020). Chapter 11: Scoping Reviews (2020 version). JBI Manual for Evidence Synthesis.

[23] Tricco A.C., Lillie E., Zarin W., O’Brien K.K., Colquhoun H., Levac D., Moher D., Peters M.D.J., Horsley T., Weeks L. (2018). PRISMA Extension for Scoping Reviews (PRISMA-ScR): Checklist and Explanation. Ann. Intern. Med..

[24] Hoffmann T.C., Glasziou P.P., Boutron I., Milne R., Perera R., Moher D., Altman D.G., Barbour V., Macdonald H., Johnston M. (2014). Better reporting of interventions: Template for intervention description and replication (TIDieR) checklist and guide. BMJ (Clin. Res. Ed.).

[25] Pollock D., Peters M.D.J., Khalil H., McInerney P., Alexander L., Tricco A.C., Evans C., de Moraes É.B., Godfrey C.M., Pieper D. (2023). Recommendations for the extraction, analysis, and presentation of results in scoping reviews. JBI Evid. Synth..

[26] Ogonowska-Slodownik A., de Lima A.A.R., Cordeiro L., Morgulec-Adamowicz N., Alonso-Fraile M., Güeita-Rodríguez J. (2022). Aquatic Therapy for Persons with Neuromuscular Diseases—A Scoping Review. J. Neuromuscul. Dis..

[27] Haddaway N.R., Page M.J., Pritchard C.C., McGuinness L.A. (2022). PRISMA2020: An R package and Shiny app for producing PRISMA 2020-compliant flow diagrams, with interactivity for optimised digital transparency and Open Synthesis. Campbell Syst. Rev..

[28] Irandoust K., Taheri M., H’Mida C., Neto G.R., Trabelsi K., Ammar A., Souissi N., Chtourou H., Nikolaidis P.T., Rosemann T. (2021). Exergaming and Aquatic Exercises Affect Lung Function and Weight Loss in Obese Children. Int. J. Sports Med..

[29] Mishe Lawson L., Kivlin N. (2022). Exploring the Effects of Swimming on Sleep Behaviors of Children with Autism Spectrum Disorder Using Single-Subject Design. Ther. Recreat. J..

[30] Adar S., Dündar Ü., Demirdal Ü., Ulaşlı A.M., Toktaş H., Solak Ö. (2017). The effect of aquatic exercise on spasticity, quality of life, and motor function in cerebral palsy. Turk. J. Phys. Med. Rehabil..

[31] Ansari S., AdibSaber F., Elmieh A. (2020). Effects of Vitamin D and/or Aquatic Exercise on IL-1β and IL-1RA Serum Levels and Behavior of Children with Autism Spectrum Disorder. Stud. Med. Sci..

[32] Ansari S., AdibSaber F., Elmieh A., Gholamrezaei S. (2021). The effect of water-based intervention on sleep habits and two sleep-related cytokines in children with autism. Sleep Med..

[33] Ansari S., Hosseinkhanzadeh A.A., AdibSaber F., Shojaei M., Daneshfar A. (2021). The Effects of Aquatic Versus Kata Techniques Training on Static and Dynamic Balance in Children with Autism Spectrum Disorder. J. Autism Dev. Disord..

[34] Ballington S.J., Naidoo R. (2018). The carry-over effect of an aquatic-based intervention in children with cerebral palsy. Afr. J. Disabil..

[35] de Araujo L.B., de Castro Silva T., Cardoso Oliveira L., Tomasetto L.C., Kanashiro M.S., Martins Braga D. (2018). Efeitos da fisioterapia aquática na função motora de indivíduos com paralisia cerebral: Ensaio clínico randomizado. Fisioter. Bras..

[36] Declerck M., Verheul M., Daly D., Sanders R. (2016). Benefits and Enjoyment of a Swimming Intervention for Youth With Cerebral Palsy: An RCT Study. Pediatr. Phys. Ther..

[37] Depiazzi J., Smith N., Gibson N., Wilson A., Langdon K., Hill K. (2021). Aquatic high intensity interval training to improve aerobic capacity is feasible in adolescents with cerebral palsy: Pilot randomised controlled trial. Clin. Rehabil..

[38] Elnaggar R.K., Alghadier M., Abdrabo M.S., Abonour A.A. (2022). Effect of a structured aqua-plyometric exercise program on postural control and functional ability in children with hemiparetic cerebral palsy: A two-arm randomized controlled trial. NeuroRehabilitation.

[39] Faraji S., Najafabadi M.G., Zandi H.G., Shaw I. (2023). Effect of aquatic therapy on motor skill and executive function in children with autism spectrum disorder. S. Afr. J. Res. Sport Phys. Educ. Recreat..

[40] Hattabi S., Forte P., Kukic F., Bouden A., Have M., Chtourou H., Sortwell A. (2022). A Randomized Trial of a Swimming-Based Alternative Treatment for Children with Attention Deficit Hyperactivity Disorder. Int. J. Environ. Res. Public Health.

[41] Hind D., Parkin J., Whitworth V., Rex S., Young T., Hampson L., Sheehan J., Maguire C., Cantrill H., Scott E. (2017). Aquatic therapy for children with Duchenne muscular dystrophy: A pilot feasibility randomised controlled trial and mixed-methods process evaluation. Health Technol. Assess..

[42] Kolachahi S.A., AdibSaber F., Zidashti Z.H., Elmieh A., Bidabadi E., Hosseinkhanzadeh A.A. (2020). Water-based training in combined with vitamin D supplementation improves lipid profile in children with ASD. Res. Autism Spectr. Disord..

[43] Marzouki H., Soussi B., Selmi O., Hajji Y., Marsigliante S., Bouhlel E., Muscella A., Weiss K., Knechtle B. (2022). Effects of Aquatic Training in Children with Autism Spectrum Disorder. Biology.

[44] Mills W., Kondakis N., Orr R., Warburton M., Milne N. (2020). Does hydrotherapy impact behaviours related to mental health and well-being for children with autism spectrum disorder? A randomised crossover-controlled pilot trial. Int. J. Environ. Res. Public Health.

[45] Naczk A., Gajewska E., Naczk M. (2021). Effectiveness of Swimming Program in Adolescents with Down Syndrome. Int. J. Environ. Res. Public Health.

[46] Perez Ramirez N.E., Cares P.N., Penailillo P.S.M. (2019). Effectiveness of Watsu therapy in patients with juvenile idiopathic arthritis. A parallel, randomized, controlled and single-blind clinical trial. Rev. Chil. Pediatr..

[47] Silva L.A.D., Doyenart R., Henrique Salvan P., Rodrigues W., Felipe Lopes J., Gomes K., Thirupathi A., Pinho R.A.D., Silveira P.C. (2020). Swimming training improves mental health parameters, cognition and motor coordination in children with Attention Deficit Hyperactivity Disorder. Int. J. Environ. Res. Public Health.

[48] Suarez-Villadat B., Luna-Oliva L., Acebes C., Villagra A. (2020). The effect of swimming program on body composition levels in adolescents with Down syndrome. Res. Dev. Disabil..

[49] Zhao P.T., Zhu G.H., Chen S., Pan Y., Chen K., Huang L., Guo L.Y. (2024). Effects of Aquatic Exercise and Floor Curling on Balance Ability and Lower Limb Muscle Strength in Children with Intellectual Disabilities: A Pilot Study in China. Children.

[50] Alaniz M.L., Rosenberg S.S., Beard N.R., Rosario E.R. (2017). The Effectiveness of Aquatic Group Therapy for Improving Water Safety and Social Interactions in Children with Autism Spectrum Disorder: A Pilot Program. J. Autism Dev. Disord..

[51] Fatorehchy S., Hosseini S.A., Rassafiani M. (2019). The effect of aquatic therapy at different levels of water depth on functional balance and walking capacity in children with cerebral palsy. Int. J. Life Sci. Biotechnol. Pharma Res..

[52] Fragala-Pinkham M.A., Smith H.J., Lombard K.A., Barlow C., O’Neil M.E. (2014). Aquatic aerobic exercise for children with cerebral palsy: A pilot intervention study. Physiother. Theory Pract..

[53] Huguet-Rodríguez M., Arias-Buría J.L., Huguet-Rodríguez B., Blanco-Barrero R., Braña-Sirgo D., Güeita-Rodríguez J. (2020). Impact of Aquatic Exercise on Respiratory Outcomes and Functional Activities in Children with Neuromuscular Disorders: Findings from an Open-Label and Prospective Preliminary Pilot Study. Brain Sci..

[54] Johnson N.L., Bekhet A.K., Karenke T., Garnier-Villarreal M. (2021). Swim Program Pilot for Children with Autism: Impact on Behaviors and Health. West. J. Nurs. Res..

[55] Martins L.G., Rocha L.P.B., Verissimo T.C.R.A., Da Silva Souza J., Prudente C.O.M., Ribeiro M.F.M. (2015). Effects of virtual rehabilitation, bobath concept, and aquatic therapy in children with cerebral palsy. Rev. Neurocienc..

[56] Ogonowska-Slodownik A., Güeita-Rodriguez J., Skomorowska K., Morgulec-Adamowicz N. (2024). Effects on Function and Enjoyment of Aquatic Therapy in Children with Cerebral Palsy: A Pilot Study in a Special Education School. Int. J. Disabil. Dev. Educ..

[57] Munn E.E., Ruby L., Pangelinan M.M. (2021). Improvements in Swim Skills in Children with Autism Spectrum Disorder Following a 5-Day Adapted Learn-To-Swim Program (iCan Swim). J. Clin. Med..

[58] Oriel K.N., Kanupka J.W., DeLong K.S., Noel K. (2016). The Impact of Aquatic Exercise on Sleep Behaviors in Children with Autism Spectrum Disorder. Focus Autism Other Dev. Disabil..

[59] Suslov V.M., Lieberman L.N., Carlier P.G., Ponomarenko G.N., Ivanov D.O., Rudenko D.I., Suslova G.A., Adulas E.I. (2023). Efficacy and safety of hydrokinesitherapy in patients with dystrophinopathy. Front. Neurol..

[60] Vodakova E., Chatziioannou D., Jesina O., Kudlacek M. (2022). The Effect of Halliwick Method on Aquatic Skills of Children with Autism Spectrum Disorder. Int. J. Environ. Res. Public Health.

[61] Yanardag M., Akmanoglu N., Yilmaz I. (2013). The effectiveness of video prompting on teaching aquatic play skills for children with autism. Disabil. Rehabil..

[62] Adin E., Pancar Z. (2023). Effect of Swimming Exercise on Respiratory Muscle Strength and Respiratory Functions in Children with Autism. Eurasian J. Med..

[63] Caputo G., Ippolito G., Mazzotta M., Sentenza L., Muzio M.R., Salzano S., Conson M. (2018). Effectiveness of a Multisystem Aquatic Therapy for Children with Autism Spectrum Disorders. J. Autism Dev. Disord..

[64] Chang Y.K., Hung C.L., Huang C.J., Hatfield B.D., Hung T.M. (2014). Effects of an aquatic exercise program on inhibitory control in children with ADHD: A preliminary study. Arch. Clin. Neuropsychol..

[65] Fruhauf A., Niedermeier M., Sevecke K., Haid-Stecher N., Albertini C., Richter K., Schipflinger S., Kopp M. (2020). Affective responses to climbing exercises in children and adolescents during in-patient treatment for mental health disorders a pilot study on acute effects of different exercise interventions. Psychiatry Res..

[66] Georgieva D., Ivanova V. (2023). Aquatic gymnastics program to improve kinesthetic manual praxis in children with Down syndrome. Pedagog. Phys. Cult. Sports.

[67] Hattabi S., Bouallegue M., Yahya H.B., Bouden A. (2019). Rehabilitation of ADHD children by sport intervention: A Tunisian experience. Tunis. Medicale.

[68] Ogonowska-Slodownik A., Kaczmarczyk K., Kokowicz G., Morgulec-Adamowicz N. (2021). Does the Aquatic Breathing Program Improve Lung Function in Adolescents with Scoliosis?. Phys. Occup. Ther. Pediatr..

[69] Zverev Y., Kurnikova M. (2016). Adapted community-based group aquatic program for developing balance: A pilot intervention study involving children and adolescents with cerebral palsy. J. Phys. Educ. Sport.

[70] Mische Lawson L., Lisk C., Carlson J., Priebe M., Shaver E., Wilson F. (2020). The Feasibility of Measuring Heart Rate of Children with Autism During Swim Lessons and Potential Health Outcomes. Ther. Recreat. J..

[71] Mische Lawson L., Little L. (2017). Feasibility of a Swimming Intervention to Improve Sleep Behaviors of Children With Autism Spectrum Disorder. Ther. Recreat. J..

[72] Conklin A., Van Wingerden A. (2023). Aquatic Cycling for a Child with Hemiplegic Cerebral Palsy: A Case Report. J. Aquat. Phys. Ther..

[73] Esmailiyan M., Marandi S.M., Darvishi M., Haghjooy Javanmard S., Amerizadeh A. (2023). The Effect of Eight Weeks of Aquatic Exercises on Muscle Strength in Children with Cerebral Palsy: A Case Study. Adv. Biomed. Res..

[74] Musiyenko O.V., Chopyk R.V., Kizlo N.B. (2020). Influence of swimming on sensory functioning, quality of life and behavior of children with autism. Health Sport Rehabil..

[75] Bekhet A.H., Johnson N., Karenke T., Van Hecke A. (2023). A Swimming Program for Children with Autism Spectrum Disorders: Assessing Critical Parameters from Caregivers’ Perspectives. Int. J. Aquat. Res. Educ..

[76] Oriel K.N., Scesa M.M., Kanupka J.W., Deardorff A.R., Grow S.E., Lane L.E., Poltonavage O.J. (2020). The Impact of an Aquatic Social Competence Program on Children with Autism Spectrum Disorder. J. Aquat. Phys. Ther..

[77] Hind D., Parkin J., Whitworth V., Rex S., Young T., Hampson L., Sheehan J., Maguire C., Cantrill H., Scott E. (2017). Aquatic therapy for boys with Duchenne muscular dystrophy (DMD): An external pilot randomised controlled trial. Pilot Feasibility Stud..

[78] Salari N., Rasoulpoor S., Rasoulpoor S., Shohaimi S., Jafarpour S., Abdoli N., Khaledi-Paveh B., Mohammadi M. (2022). The global prevalence of autism spectrum disorder: A comprehensive systematic review and meta-analysis. Ital. J. Pediatr..

[79] Date S., Munn E., Frey G.C. (2024). Postural balance control interventions in autism spectrum disorder (ASD): A systematic review. Gait Posture.

[80] Ruggeri A., Dancel A., Johnson R., Sargent B. (2020). The effect of motor and physical activity intervention on motor outcomes of children with autism spectrum disorder: A systematic review. Autism.

[81] Ayano G., Demelash S., Gizachew Y., Tsegay L., Alati R. (2023). The global prevalence of attention deficit hyperactivity disorder in children and adolescents: An umbrella review of meta-analyses. J. Affect. Disord..

[82] Abdelnour E., Jansen M.O., Gold J.A. (2022). ADHD Diagnostic Trends: Increased Recognition or Overdiagnosis?. MoMed.

[83] Ogonowska-Slodownik A. (2022). The Use of Aquatic Environment for Children with Disabilities. Palaestra.

