# Supplementary material for: Aquatic Therapy in Children and Adolescents with Disabilities: A Scoping Review
Source: Children (Basel). 2024 Nov 20;11(11):1404. doi: 10.3390/children11111404 (PMC11593235; doi:10.3390/children11111404)
Supplement: Supplementary file 1 [file children-11-01404-s001.zip › children-3315459-supplementary.pdf]

Table S1: Full Search Strategy for MEDLINE.

MEDLINE, n=214

Interface: Ovid

Search Screen: Advanced Search

Search modes: Boolean/Phrase

Database: Ovid MEDLINE(R) ALL <1946 to February 15, 2024>

#	**Query**	**Results February 16, 2024**
1	Hydrotherapy/	2,642
2	Aquatic therapy/	29
3	adapted aquatic*.mp.	8
4	(aqua-plyometric* or AquaPlyo).mp.	4
5	Halliwick.mp.	23
6	Watsu.mp.	13
7	Ai chi.mp.	33
8	Bad Ragaz Ring.mp.	6
9	(water shiatsu or watershiatsu).mp.	5
10	(water adj exercise*).mp.	227
11	(water-based adj3 exercise*).mp.	214
12	(aquatic* adj3 exercise*).mp.	673
13	(aquatic* adj3 intervent*).mp.	131
14	(aquatic* adj3 therap*).mp.	325
15	(aquatic* adj3 physical therap*).mp.	36
16	(aquatic* adj3 physiotherap*).mp.	38
17	(aquatic* adj3 rehab*).mp.	83
18	(swim* adj3 therap*).mp.	83
19	(swim* adj3 rehab*).mp.	38
20	swimming.mp.	43,598
21	or/1-20 [aquatic therapy concept]	47,048

22	disab*.mp.	418,766
23	exp neurodevelopmental disorders/	213,599
24	adhd.mp.	33,358
25	Adrenoleukodystroph*.mp.	2,701
26	Angelman syndrome.mp.	2,030
27	Asperger*.mp.	2,943
28	attention deficit.mp.	50,230
29	(autism or autistic).mp.	73,332
30	blind.mp.	305,326
31	(cerebellar adj ataxia*).mp.	9,763
32	cerebral palsy.mp.	32,892
33	Coffin-Lowry syndrome.mp.	250
34	Cri-du-Chat.mp.	834
35	De Lange Syndrome.mp.	1,194
36	Dyskinesias.mp.	6,497
37	deaf*.mp.	54,461
38	developmental disorder*.mp.	11,115
39	down* syndrome.mp.	33,900
40	(ehlers adj danlos).mp.	5,099
41	fetal alcohol.mp.	6,629
42	fragile x.mp.	9,325
43	(guillain adj barre*).mp.	12,977
44	hearing loss*.mp.	82,001
45	Hydrocephalus.mp.	38,649
46	Lesch-Nyhan Syndrome.mp.	1,442
47	Lissencephalies.mp.	240
48	(Menkes adj3 syndrome).mp.	1,104
49	(mental* adj retard*).mp.	38,144

50	Motor skills disorder*.mp.	3,320
51	Movement Disorder*.mp.	36,280
52	(Mucopolysaccharidosis or san Filippo syndrome or sanfilippo syndrome).mp.	7,225
53	muscular dystroph*.mp.	34,965
54	neurodevelopmental disorder*.mp.	20,483
55	parapleg*.mp.	24,309
56	prader-willi.mp.	4,610
57	rett syndrome.mp.	4,321
58	Rubinstein-Taybi.mp.	839
59	spina bifida.mp.	9,910
60	spinal cord injur*.mp.	60,715
61	Tourette*.mp.	6,794
62	(Trisomy adj3 syndrome).mp.	3,115
63	WAGR Syndrome.mp.	221
64	Williams Syndrome.mp.	2,555
65	Wolf-Hirschhorn Syndrome.mp.	573
66	visual* impair*.mp.	19,041
67	or/22-66 [disabilities concept]	1,284,446
68	exp Child/	2,189,209
69	exp child, preschool/	996,581
70	child*.mp.	2,814,926
71	pre-school*.mp.	6,026
72	school age*.mp.	29,578
73	(girl* or boy*).mp.	281,701
74	paediatric*.mp.	89,410
75	pediatric*.mp.	464,461
76	juvenile*.mp.	108,548

77	youth*.mp.	110,425
78	(young adj2 (person* or people)).mp.	43,056
79	teen*.mp.	36,846
80	adolescen*.mp.	2,331,208
81	or/68-80 [children and adolescents concept]	4,243,407
82	and/21,67,81	441
83	exp animals/ not humans.sh.	5,198,013
84	82 not 83 [limit our animal studies]	403
85	limit 84 to yr="2012-current"	214
